# Les psychoses de l’épileptique: approche clinique à propos d'un cas

**DOI:** 10.11604/pamj.2014.17.265.4158

**Published:** 2014-04-12

**Authors:** Nada Charfi, Dorsaf Trigui, Jihène Ben Thabet, Nasreddine Zouari, Lobna Zouari, Mohamed Maalej

**Affiliations:** 1 Service de Psychiatrie « C », CHU Hédi Chaker, 3029 Sfax, Tunisie

**Keywords:** Clinique, classification, comorbidité, diagnostic, épilepsie, psychose, clinic, classification, comorbidity, diagnosis, epilepsy, psychosis

## Abstract

Pour discuter les liens entre épilepsie et psychose, les auteurs rapportent l'observation d'une fille âgée de 22 ans, traitée pour épilepsie fronto-temporale, adressée en psychiatrie pour des hallucinations sensorielles et cénesthésiques et un délire mystique et d'influence, apparus secondairement et non améliorés par le traitement antiépileptique. Les symptômes psychotiques, chez l’épileptique, peuvent entrer dans le cadre de psychoses intercritiques, post-critiques ou alternatives. Pour le cas rapporté, les symptômes psychotiques étaient intercritiques et chroniques. Il s'agissait vraisemblablement d'une psychose schizophréniforme. Dans ce type de psychose, une indifférence affective et une restriction des activités sont rarement rencontrés, alors que les fluctuations rapides de l'humeur sont fréquentes. Les thématiques délirantes sont assez souvent mystiques, alimentées par des hallucinations auditives et par des hallucinations visuelles inhabituelles. Les troubles négatifs sont rares. Les psychoses épileptiques n'ont pas été identifiées comme des entités nosographiques dans les systèmes de classification psychiatrique (DSM-IV et CIM-10), ce qui pose un problème de reconnaissance de ces troubles. Une collaboration entre psychiatre et neurologue devient ainsi nécessaire pour mieux comprendre cette comorbidité complexe, éviter les erreurs diagnostiques et optimiser la prise en charge.

## Introduction

L’épilepsie représente encore de nos jours un véritable problème de santé mentale; car si la plupart des épilepsies sont curables, leurs conséquences psychopathologiques demeurent souvent importantes et de prise en charge complexe [1]. L'association de l’épilepsie avec les troubles psychotiques, en particulier, est connue depuis longtemps [2]. Ces troubles ont été individualisés sous des appellations diverses: psychose per-critique, psychose post-critique, psychose alternative et psychose schizophréniforme. Se pose alors la question de la relation entre ces deux types de troubles: s'agit-il d'une association fortuite ou y a-t il un rapport spécifique entre les deux et quelles sont les implications thérapeutiques?À partir de l'analyse d'une observation mettant en relief les enjeux cliniques et thérapeutiques de la comorbidité épilepsie-troubles psychotiques, une revue bibliographique a été réalisée pour étudier les rapports entre ces deux types de troubles.

## Patient et observation

Mademoiselle S.J, âgée de 20 ans, sans antécédents familiaux de psychose ou d′épilepsie, a été adressée en psychiatrie par un médecin neurologue pour des perceptions anormales, sensorielles (auditives et visuelles) et cénesthésiques (sensations de pénétration vaginale), non améliorées par le traitement antiépileptique. Il s'agissait d'une étudiante en droit, célibataire et vivant avec sa mère depuis le divorce précoce de ses parents. La jeune fille était décrite par sa mère comme une personne laborieuse, appliquée et bien insérée socialement. Le début des troubles morbides de l'intéressée remontait à deux ans, marqué par l'apparition de crises nocturnes faites de réveils brusques accompagnés de dystonies au niveau des membres inférieurs. Ces crises étaient de durée brève, se répétant de façon stéréotypée et suivies d'une amnésie partielle des faits. Initialement, ces évènements nocturnes ont été sous estimés par la famille de la patiente et mis sur le compte d'un trouble du sommeil transitoire dû au stress des examens. Secondairement, soit quelques mois après, il y a eu apparition de perceptions auditives à contenu injurieux et angoissant. Elle entendait une voix terrifiante qui lui disait « l'amour est un pêché puni par Dieu, tu finiras en enfer et ton bien-aimé va bientôt mourir ». Ces perceptions évoluaient de façon paroxystique, précédées de manifestations neuro-végétatives faites de douleur abdominale, de sensation d’épigastralgie ascendante et de nausées, et suivies de perte de contact. Depuis, la patiente est devenue très angoissée, n'arrivait plus à rester seule dans sa chambre et éprouvait des difficultés pour s'endormir le soir. Elle est devenue absorbée par ces voix étranges; ce qui l'a rendue distraite la plupart du temps et a retenti sur son rendement scolaire. Son état s'est aggravé par l'apparition de sensations de pénétration vaginale et de voix lui disant « qu'elle a été violée et qu'elle n’était plus vierge ». Tourmentée par l’éventualité qu'elle ne fût plus vierge, elle a consulté un gynécologue qui l'a rassurée quant à sa virginité et l'a adressée par la suite à un médecin neurologue. Une IRM cérébrale s'est révélée normale tandis que l'EEG de veille a été de type comitial (décharges de pointes frontales et temporales prédominantes à droite). La patiente a alors été mise sous carbamazépine à la dose de 600 mg/jour.

L’évolution sous traitement a été marquée par une nette diminution de la fréquence des crises dystoniques nocturnes et neurovégétatives et une normalisation de l'EEG de contrôle. Cependant, les perceptions auditives se sont accentuées pour devenir permanentes et le tableau s'est enrichi par d'autres éléments incluant un automatisme mental, un délire à thèmes d'influence et mystique (le nom de Dieu revenait à plusieurs reprises dans son discours) et des hallucinations visuelles terrifiantes. Ces manifestations survenaient sans aucun signe d'altération de l’état de conscience. C'est là que la patiente a été adressée en psychiatrie. Il est à signaler également qu’à l′âge de 9 mois, notre patiente eut des convulsions fébriles ayant nécessité une hospitalisation prolongée. Par la suite, le diagnostic d’épilepsie fut porté et elle a été mise sous un traitement anticomitial. Ce traitement a été arrêté au bout de douze mois, de façon intempestive, par sa mère, craignant ses effets indésirables. Ces renseignements cliniques nous ont été fournis par cette dernière après plusieurs consultations. Il s'avère donc que l'histoire de l’épilepsie de l'intéressée remonte à son enfance.

Pour les troubles décrits ci-dessus, le diagnostic d’épilepsie partielle ne pouvait pas expliquer la totalité du tableau clinique; l'hypothèse d'un trouble psychotique comorbide en l'occurrence une psychose schizophréniforme de l’épilepsie était vraisemblable. Ceci avait justifié sa mise sous antipsychotique atypique (la rispéridone: 2mg /jour) en association avec son traitement antiépileptique. L’évolution était assez bonne, avec atténuation des perceptions anormales et mise à distance des convictions délirantes.

## Discussion

Chez cette patiente, les crises épileptiques qui étaient survenues durant la petite enfance, sont réapparues vers l’âge de 20 ans avec une autre présentation clinique. Les crises nocturnes faites d’éveils paroxystiques et de dystonies correspondent vraisemblablement à des crises d’épilepsie frontale nocturne. Dans ce type d’épilepsie, souvent méconnu par le patient et son entourage, les crises surviennent exclusivement pendant le sommeil et posent parfois un problème de diagnostic différentiel avec les manifestations motrices paroxystiques liées à un trouble du sommeil [3]. Alors que les symptômes auditifs et neurovégétatifs avec les phénomènes expérientiels du début, évoluant de façon paroxystique, orientent vers une épilepsie temporale. Le diagnostic global était celui d'une épilepsie fronto-temporale complexe, vu qu'il y avait la notion de perte de contact et d'amnésie partielle post-critique. Ce diagnostic a été retenu sur la base des différents arguments anamnestiques, cliniques et électriques (pointes ondes frontales et temporales). Toutefois, en dehors de ces crises épileptiques stéréotypées, l'enrichissement ultérieur de la symptomatologie par les hallucinations sensorielles (auditives, visuelles et cénesthésiques) non concomitantes aux crises et non reconnues comme pathologiques par notre patiente, les idées délirantes à thèmes mystique et d'influence et l'automatisme mental, sans trouble de la conscience, évoquaient une psychose associée à l’épilepsie. Tout l'enjeu est dans la reconnaissance de cette comorbidité malgré le polymorphisme sémiologique de cette épilepsie partielle fronto-temporale et l'atypicité des troubles psychotiques développés par notre patiente au cours de l’évolution. En effet, les hallucinations cénesthésiques de viol, qui constituent une des originalités de notre observation, ne sont pas habituelles dans les crises partielles et devraient attirer l'attention des neurologues vers un trouble psychotique associé. Le délire d'influence, autre originalité de l'observation, est rare dans la psychose épileptique, contrairement au délire mystique. Mais l'existence d'autres éléments comme l'automatisme mental et le syndrome hallucinatoire riche rendent ce diagnostic très probable. Ces données cliniques renvoient à la schizophrénie, cependant peut-on parler de schizophrénie chez le sujet épileptique et quels sont, le cas échéant, les spécificités de cette schizophrénie ‘ Les données de la littérature suggèrent que les patients épileptiques ont un risque de développer une psychose 6 à 12 fois plus important que dans la population générale [4, 5] et ce risque s'avère plus important dans le cas d’épilepsie temporale [6]. Reste à savoir, dans notre cas, de quel type de psychose épileptique s'agit-il? Une tentative de classification des psychoses de l’épileptique, tenant compte de la durée de l’épisode psychotique et de la chronologie de son apparition par rapport aux crises épileptiques ainsi que du niveau de conscience, distingue trois types [2]: les psychoses épisodiques où la conscience est souvent altérée (psychose critique, confusion post-critique, psychose post-critique), les psychoses permanentes ou chroniques où la conscience est conservée (psychoses intercritiques shizophréniformes) et les psychoses alternatives, où le niveau de conscience est variable.

### 1. Les psychoses critiques et péri-critiques

Au cours de certaines crises épileptiques partielles, des manifestations d'allure psychotique comme la pensée forcée, l’état de rêve, les hallucinations ou les illusions simples ou complexes, peuvent se voir. D'ailleurs, la patiente que nous avons présentée a vécu également des troubles critiques au début de l’évolution de son épilepsie, faits d'hallucinations auditives angoissantes et des illusions de viol. Ces manifestations seront facilement rattachées à l'origine épileptique devant leur durée brève, leur caractère stéréotypé et surtout leur vécu différent par les patients [2]. La confusion post-critique ne pose habituellement pas de problème diagnostique étant donnée sa brièveté et son lien évident avec la crise d’épilepsie. En revanche, dans l’état de mal épileptique, elle se prolonge dans le temps et pourrait ainsi évoquer à tort une affection psychiatrique. La confusion reste cependant caractérisée par des réactions de défense mal adaptées, un oppositionnisme, des comportements stéréotypés, une agitation et une anxiété. L’évolution spontanément favorable, en quelques heures, et les antécédents d’épilepsie permettront de rétablir le diagnostic, a posteriori le cas échéant. L'EEG constitue toujours l'outil diagnostique principal puisqu'il s'avère constamment anormal: il montre soit une activité de pointes témoignant du caractère critique de l’épisode, soit des anomalies lentes de distribution et d'amplitude variables orientant vers un état post-critique prolongé [7]. Les psychoses critiques ou péri-critiques sont habituellement brèves et limitées à la durée de la crise ou de l’état de mal épileptique. Néanmoins, leur présentation clinique sous forme d'authentiques états confusionnels entrant dans le cadre de la crise rend la qualification « psychotiques » de ces épisodes comme une sorte de glissement sémantique fortement contestable [1].

### 2. Les psychoses post-critiques

La psychose post-critique est une entité d'individualisation assez récente [8]. Elle est caractérisée par une chronologie précise: la survenue d'une crise ou d'une salve de crises, confusion post-critique habituelle, retour à une conscience normale, intervalle de complète lucidité, puis apparition soudaine d'un trouble psychotique de durée brève évoluant en pleine conscience ou avec une conscience discrètement altérée [9]. Elle apparaît essentiellement chez des patients présentant une épilepsie partielle réfractaire, évoluant depuis au moins 10 ans [7]. Il s'agit le plus souvent de crises partielles complexes temporales et de crises secondairement généralisées [8, 10] avec volontiers des anomalies bitemporales intercritiques à l'EEG [11]. Le trouble psychotique survient après une série de crises très rapprochées, typiquement sous la forme d'une salve de crises. Le début est brutal, après un intervalle libre de 24 heures à 1 semaine à l'issue de la dernière crise. Le tableau psychiatrique est caractérisé par un délire aigu associant en proportions variables: des troubles de l'humeur, souvent au premier plan, des hallucinations auditives, un délire à thématiques religieuses ou de grandeur, un délire de persécution [10]. L'EEG critique est normal et l'EEG intercritique montre la fréquence de foyers bitemporaux indépendants [1]. Les patients atteints ont plus fréquemment des antécédents psychiatriques quand ils sont comparés à des épileptiques appariés non psychotiques [9]. L’évolution est spontanément résolutive au bout d'une semaine au maximum [11], avec une amnésie de l’épisode et possibilité de récidives ultérieures sous forme d’épisodes identiques. Dans certains cas sévères, un traitement psychotrope s'avère indispensable pour gérer les troubles du comportement.

### 3. Les psychoses alternatives

Contrairement à la psychose post-critique qui fait suite à une période d'activité de la maladie épileptique, la psychose alternative apparaît quand les crises disparaissent suggérant un phénomène de balancement [2]. La première description de ce phénomène a été faite par Landolt qui, dès 1953, a constaté une corrélation entre l’évolution du processus psychotique et les modifications de l'EEG: le foyer d'anomalies paroxystiques qui était actif avant et après l’épisode psychotique, disparaissait pendant cet épisode [12]. Il a désigné cette normalisation de l'EEG par le terme « normalisation forcée de l'EEG »: concept caractérisé par le fait que, avec l'apparition de l’état psychotique, l'EEG s'améliore ou devient même entièrement normal comparé aux tracés précédents. Cet état, qui peut durer plusieurs semaines, peut être déclenché par la mise en route d'un traitement antiépileptique et interrompu par une sismothérapie. Tellenbach [13] a associé à ce concept de « normalisation forcée » qui est uniquement éléctroencéphalographique, le terme de « psychose alternative/alternante » pour insister sur la disparition des crises à l'occasion d'un épisode psychotique, quelque soit le tracé EEG. Ce type de psychose est rare. Généralement, les épisodes sont brefs mais parfois ils peuvent se prolonger sur plusieurs semaines. Il existe souvent une phase prodromale commune avec tendance à l'anxiété, doléances hypochondriaques et insomnie. Puis, à la phase d’état, on trouve un délire à mécanisme essentiellement imaginatif et une conscience légèrement altérée. Des symptômes hystériques, dépressifs, maniaques, dysphoriques et des préoccupations hypocondriaques peuvent compléter le tableau clinique. La psychose alternative peut se rencontrer dans les épilepsies aussi bien partielles que généralisées, mais la présence simultanée de crises tonico-cloniques et de crises non convulsives accompagnées d'une altération de la conscience (soit absences, soit crises partielles complexes) semble nécessaire [14].

### 4. Les psychoses inter-critiques shizophréniformes

Les psychoses inter-critiques ou inter-ictales chroniques ont une présentation clinique évocatrice chez les patients épileptiques, sous forme de schizophrénie [15, 16]. Ey a introduit le terme de psychose schizo-comitiale [17] pour décrire ce type de psychose, alors que dans la littérature anglo-saxonne, les cas rapportés ont été dénommés « psychoses schizophréniformes de l’épilepsie (schizophrenia-like psychoses of epilepsy) » [18, 19]. Sur le plan clinique, différentes formes peuvent s'observer. On peut observer une forme paranoïaque [2], où les patients ayant des traits de personnalité sensitive ou soupçonneuse, vont développer des hallucinations auditives avec une thématique souvent interprétative. Toutefois, on ne peut parler de délire paranoïaque du fait d'un manque de systématisation. On peut observer aussi une forme paranoïde [2] qui est considérée comme une véritable psychose schizophrénique de l’épilepsie. Sur le plan sémiologique, quelques nuances ont été relevées [9]: dans les psychoses épileptiques, une indifférence affective et une restriction des activités sont rarement rencontrés, alors que les fluctuations rapides de l'humeur sont fréquentes. Les thématiques délirantes sont assez souvent mystiques, alimentées par des hallucinations auditives et par des hallucinations visuelles inhabituelles. Les troubles négatifs sont rares. L’épilepsie débute avant l’âge de 10 ans et un intervalle d'environ14 ans sépare le début de l’épilepsie de la psychose [9]. Il n'y a pas de personnalité prémorbide de type schizoïde ni d'antécédents familiaux de schizophrénie. L'existence d'une épilepsie temporale est considérée comme le facteur de risque principal, indépendamment de la gravité intrinsèque de l’épilepsie mesurée par la fréquence des crises. Enfin, l’évolution des psychoses épileptiques paraît moins déficitaire que celle des schizophrénies endogènes.

On peut ainsi rapprocher le cas de la patiente étudiée de cette entité nosographique: la psychose schizophréniforme. En effet, Melle S.J avait présenté, après des années d’évolution de son épilepsie de type fronto-temporale, un délire paranoïde chronique à thème mystique et d'influence et à mécanisme hallucinatoire (des hallucinations auditives, visuelles et cénesthésiques). L'absence de personnalité prémorbide de type schizoïde, d'antécédents familiaux de schizophrénie, d'altération du cours de sa pensée et de retentissement important sur son fonctionnement social et scolaire corroborent encore cette hypothèse diagnostique.

## Conclusion

L'observation clinique présentée et la revue de la littérature illustrent la variété des troubles psychotiques pouvant être associés à l’épilepsie au cours de son évolution et leurs spécificités. Cette comorbidité suppose l'existence d'une relation causale entre épilepsie et psychose, dont l'explication reste hypothétique. Les psychoses épileptiques, bien qu'elles présentent des spécificités cliniques et évolutives, n'ont pas été identifiées comme des entités nosographiques dans les systèmes de classification psychiatrique (DSM-IV et CIM-10), ce qui pose un problème de reconnaissance de ces troubles. Par conséquent, une collaboration entre psychiatre et neurologue devient nécessaire pour une description précise du trouble psychotique, d'une part, et de l’épilepsie, d'autre part, afin de mieux comprendre cette comorbidité complexe, d’éviter les erreurs diagnostiques et d'optimiser la prise en charge.
